# Detection of alkanolamines in liquid cement grinding aids by HPLC coupled with evaporative light scattering detector

**DOI:** 10.3906/kim-2007-8

**Published:** 2021-04-28

**Authors:** Cheng LI, WenFang SONG, YuBai SHI, ZhiWei QING

**Affiliations:** 1 Department of Health Inspection and Quarantine Technology, Faculty of Medical Technology, Ningbo College of Health Science, Ningbo China; 2 Department of Asset Management Office, Zhejiang Pharmaceutical College, Ningbo China

**Keywords:** Diethanol isopropanolamine, triisopropanolamine, HPLC-ELSD; triethanolamine, cement grinding aids

## Abstract

Triethanolamine (TEA), triisopropanolamine (TIPA), diethanol isopropanolamine (DEIPA) are necessary cement additives, and knowing their contents is needed for quality control and also to understand final properties of the cement. Whether it is the production of grinding aids, technical research and development or application research all involve the detection of grinding aids. This work developed a simple analytical technique for the simultaneous analysis of these alkanolamines in liquid cement grinding aids using high-performance liquid chromatography (HPLC) combined with evaporative light scattering detection (ELSD). HPLC was conducted by an XBridge C18 column (with dimensions 4.6 × 250 mm and 5 µm particles) using methanol and 0.1% trichloroacetic acid as mobile elution phases. The ELSD sprayer and drift tube temperatures were 60 ºC and 90 ºC, respectively. HPLC-ELSD developed in this work demonstrated 1) high sensitivity with limits of detection for the three analytes are 0.15, 0.54, 1.04 µg/mL; 2) good linearity with correlation coefficients equal to 0.997–0.999 over the tested concentration range; 3) excellent repeatability with intra- and interday coefficient of variation (CV) below 2.71% and 4, satisfactory accuracy with recovery in the 95.5%–100.8% range. This novel method is a powerful, time- and costeffective tool for alkanolamine analyses and quality control.

## 1. Introduction

The utilisation of grinding aids in cement manufacturing increases grinding efficiency, reduces the production cost and energy consumption. It also affects particle-size distribution, improves compressive and flexural strengths of cement, reduces greenhouse gases, increases cement admixture levels, etc. 

Cement grinding aids include compounds groups such as alkanolamines, glycols, or phenols. Organic alkanolamines such as TEA, DEIPA, and TIPA are very efficient grinding aids and widely used. Often, for quality control and to predict and assess final product properties, contents of these alkanolamines in liquid raw materials of cement grinding aids have to be known [1–4]. However, these alkanolamines are very polar molecules and are very similar structurally (they differ in the CH3 group amount and position). Thus, because of this and because these molecules do not possess chromophore, fluorophore, or electroactive groups, their chromatographic separation as well as quantification and detection are very challenging and difficult. 

Most of the reported methods suitable for alkanolamine analyses are from other industrial applications and not from the cement industry. Typical methods of alkanolamine detection in various specimens include gas chromatography (GC), ion exclusion chromatography (IEC), capillary electrophoresis (CE), and liquid chromatography (LC),main details of which are described as follows. Many of the recently reported GC methods provides a feasible method for detecting alkanolamines usingspecific columns that allow direct aqueous injections [5].The CE[6,7] and IEC [8]methods were also used for determination of alkyl- and alkanolaminesin drinking and natural waters samples, but alkanolamines have only weak UV absorbance, leading to a low sensitivity.The high-performance liquid chromatography (HPLC) coupled with post-column fluorescence derivatisation (FLD)[9,10] require complex sample derivatisation preparation and is time consuming. The liquid chromatography coupled with mass spectrometry (LC-MS/MS)[11,12] has unique identification capabilities when the chromatographic peaks are not completely separated, but it requires expensive equipment which it is not suitable for routine quality control in regular laboratories.

Therefore, this study focuses on overcoming these problems by using a combination of HPLC and ELSD. ELSD is a general-purpose detector that can detect organic substances without ultraviolet absorption, and has a very important application in chromatographic analysis [13–16]. To our knowledge, this study was the first time that HPLC method had been applied for the determination of TEA, TIPA and DEIPA in liquid cement grinding aids. 

The main purpose of this study was to develop an accurate and robust method suitable for routine quality control of cement grinding aids. First, an appropriate concentration standard solution of three composition was chosen and tested. The composition and flow rate of mobile phase of the method was optimised. Then, the analytical performances of the developed method were evaluated in terms of sensitivity, linearity, repeatability and accuracy. 

## 2. Materials and methods

### 2.1. Reagents and samples 

HPLC grade methanol was purchased from Tedia Co. (Fairfielr, OH, USA). TEA (97% of purity), DEIPA (94%, containing stereo and chiral isomers), and TIPA (95%, also containing isomers) were acquired from Merck Chemicals Co. (Shanghai, China). Trichloroacetic acid of analytical grade was purchased from Aladdin Reagent Co (Shanghai, China). All other reagents were at least of analytical grade. Deionised water was prepared using a Millipore system. The raw materials of cement grinding aids samples were from KLC and Cement Additives Co. (Beijing, China). 

### 2.2. Stock and working standard solution preparation 

To prepare a stock standard solution, TEA, DEIPA and TIPA were weighed (with 0.1 mg accuracy), placed into a 100 mL flask, mixed well by ultrasonicating for 10min and made up to 100 mL. The final concentrations of TEA, DEIPA, and TIPA were 5068, 5660, and 6872 µg/mL, respectively. This stock standards solution was then diluted 2, 4, 10, 20, and 40 times using 0.1% trichloroacetic acid solution-methanol (9:1, v/v) for the five working standard solutions. 

### 2.3. Sample preparation

The raw materials of cement grinding aids samples with known amounts of grinding aids were chosen to match the range of the corresponding alkanolamines in the external standard solution. Thus, a 0.1g sample of liquid cement grinding aids was weighed into a 100 mL volumetric flask, and then dissolved and extracted with 100 mL of 9:1 (v/v) 0.1% trichloroacetic acid: methanol for 10 min under ultrasonic vibration. The resulting solution was diluted by a factor 4 or 20 to obtain contents close to the median concentration of the calibration solutions. An aliquot of 20 µL of the sample solution was injected for analysis.

### 2.4. Instrumental characterisation 

Shimadzu LC-20AT HPLC consisting of a Chromachem ELSD detector (fabricated by ESA Inc., Chelmsford, MA, USA) and an XBridge C18 column (4.6 x 250 mm in size with 5 µm particles) fabricated by Waters Inc. (Milford, MA, USA) was used for all analyses. ELSD parameters were 60 ºC sprayer temperature, 90 ºC drift tube temperature, attenuation setting equal to 5, and 28 psi N2 pressure. 

During HPLC, flow rate, column temperature, and sample injection volume were 1 mL/min, 30 ºC and 20 µL, respectively. The mobile phase consisted of methanol (A) and 0.1% trichloroacetic acid (B). The gradient elution conditions are shown in Table 1. 

**Table 1:** Gradient elution conditions.

Timemin	methanol (phase A)%	0.1% trichloroaceticacid (phase B) %
0.00	10	90
12.00	10	90
12.01	50	50
15.00	80	20
20.00	80	20
20.01	10	90
30.00	10	90

## 3. Results and discussion

### 3.1. HPLC condition optimisation

**Figure 1:**
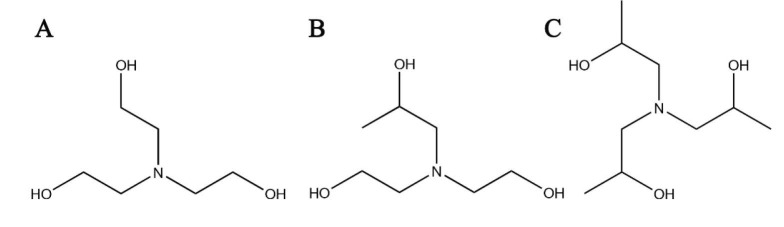
Structures of (A) triethanolamine (TEA), C_6_H_15_NO_3_, MW (molecular weight) 149.18 g/mol. (B) diethanol isopropanolamine (DEIPA), C7H17NO3, MW 163.21 g/mol. (C) triisopropanolamine (TIPA), C_9_H_21_NO_3_, MW 191.3 g/mol.

**Figure 2:**
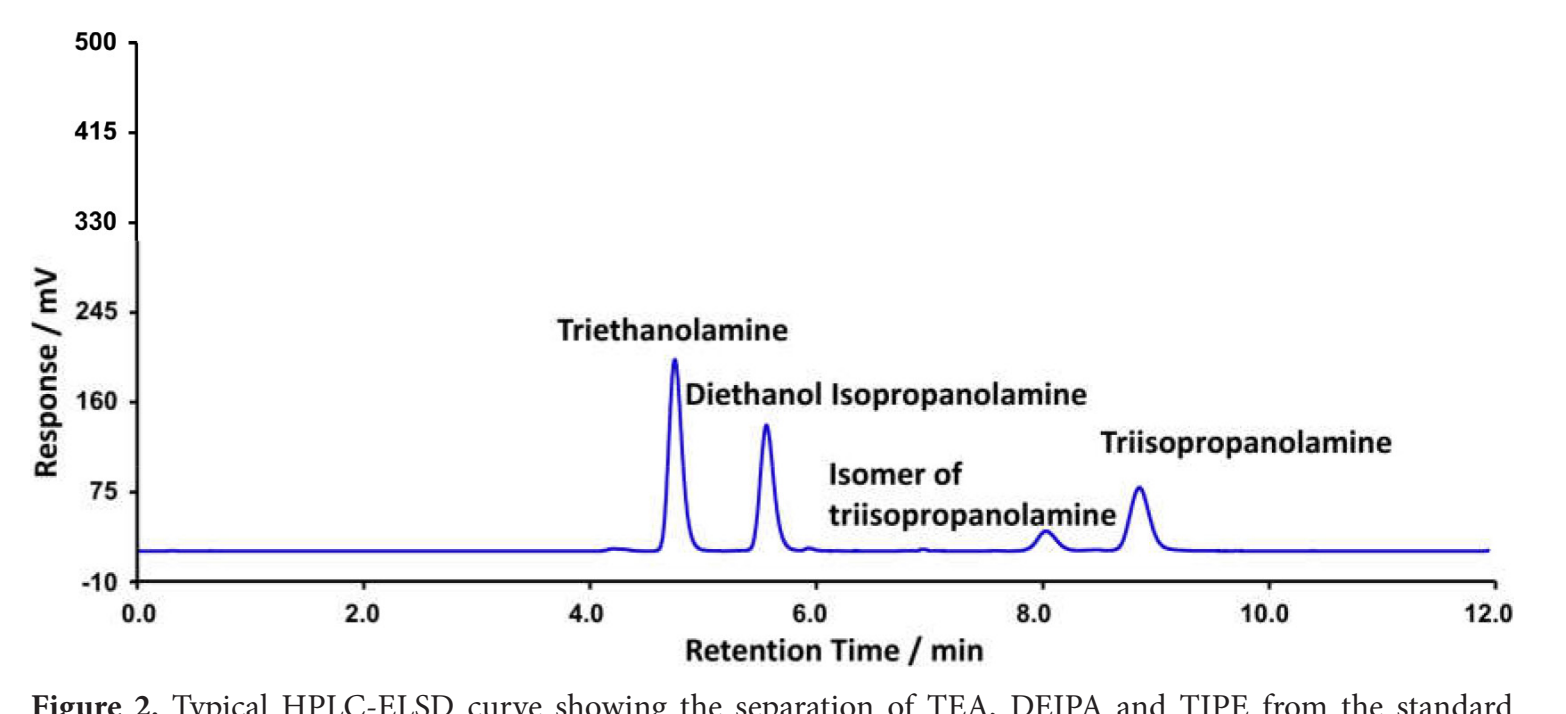
Typical HPLC-ELSD curve showing the separation of TEA, DEIPA and TIPE from the standard solution.

After the mobile phase composition and elution time optimisation, baseline separation between TEA, DEIPA, and TIPA was obtained. All three alkanolamines segregated entirely in a mobile phase containing 10% of methanol and 90% of trichloroacetic acid withing in 12 min. The column was cleaned using the same elution solvent prior to every injection. A typical HPLC-ELSD chromatogram is shown in Figures 1 and 2.

**Figure 3:**
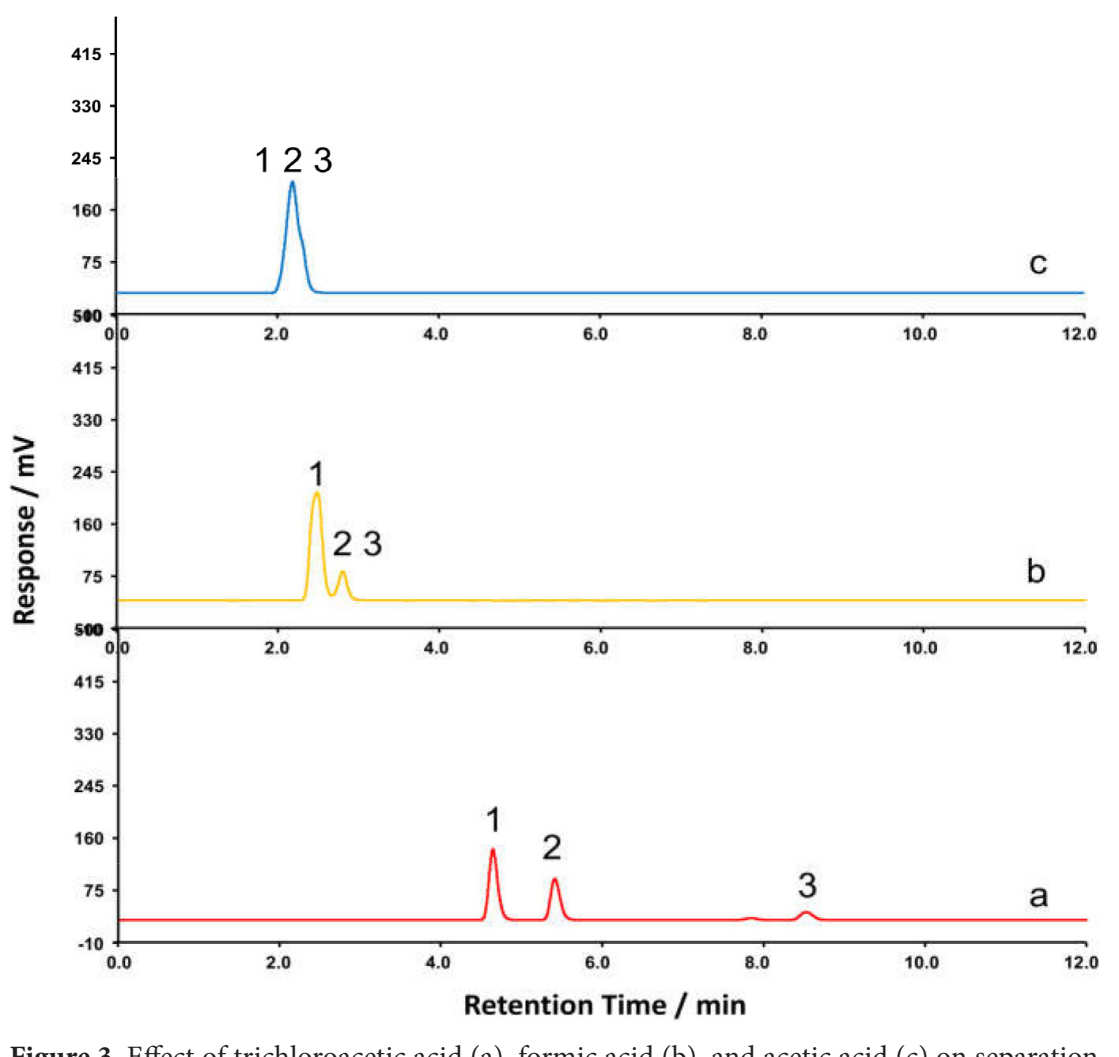
Effect of trichloroacetic acid (a), formic acid (b), and acetic acid (c) on separation efficiency; other conditions: mobile phase, methanol /acid solution modifier (10:90, v/v).1. TEA. 2. DEIPA. 3. TIPA.

All three alkanolamines used in this work (TEA, DEIPA, and TIPA) are basic because they contain one N atom. The basic functional groups are expected to produce tailing peaks because of their strong interaction with column silanol groups. Low pH values can improve chromatographic peak shape because free silanol group Si-OH on the HPLC column will not be in their ionised form Si-O(minus). HPLC-ELSD performed at low pH values allowed analysis and comparison of various acids (trifluoroacetic, acetic, formic, etc). The results showed that usage of the mobile phase consisting of methanol and trichloroacetic acid mixture resulted in better resolution and detection sensitivitythan when methanol-acetic acid or methanol-formic acid mixtures were used as mobile phases (see Figure 3).

### 3.2. Selection of evaporative light scattering detection conditions

Drift tube temperature is one of the most critical ELSD parameters. At high drift tube temperatures, the analytes might completely evaporate or even be destroyed and become undetectable. At low drift tube temperatures, the baseline noise might be too severe due to slowly and not fully evaporating mobile phase. Thus, after we tested ELSD response (and its noise level) at drift tube temperatures in the 60–100 ºC range, we established that the optimum temperature was 90 ºC.

The sprayer temperature affects the size and the uniformity of the solvent droplets, which is important for the ELSD sensitivity and reproducibility. To test how sprayer temperature influenced the results, we increased the sprayer temperature by 5 ºC starting at 30 ºC until the allowable baseline noise level appeared. At 60 ºC, a constant repeatable atomisation and acceptable baseline noise (<1 mV) were obtained. This sprayer temperature was used for all further experiments. 

### 3.3. Calibration curves 

Data obtained from the calibration curves are shown in Table 2. ELSD response increased as analyte concentration increased. Linear calibration curves (with r ≥ 0.997) were obtained when the logarithm of peak areas was plotted as a function of the logarithm of the concentration of each analyte. 

**Table 2:** Numerical equations for the calibration curves as well as LOD and LOQ data for TEA, DEIPA and TIPA analyzed by HPLC-ELSD.

Analytes	Calibration curvesa	Correlation factor	Linearity rangeµg/mL	LODµg/mL	LOQµg/mL
TEA	y = 1.4716x + 0.3822	0.999	12.67–253.4	0.15	0.51
DEIPA	y = 1.5867x + 0.1987	0.997	14.15–283.0	0.54	1.77
TIPA	y = 1.4679x - 0.0896	0.998	17.18–343.6	1.04	3.43

a Y: the logarithm of the peak area; X: the logarithm of the concentration.

### 3.4. Limit of detection and limit of quantification

The ranges of the limit of detection (LOD) for the three alkanolamines were 0.15 to 1.04 µg/mL. The limit of quantification (LOQ) varied from 0.51 to 3.43 µg/mL. The LOD and LOQ were obtained at the signal-to-noise ratios equal to 3 and 10, respectively.

### 3.5. Precision 

The intraday and interday variations were analysed for the precision estimation of the method developed in this work. For this purpose, we performed six tests for each analyte in one day. The average was calculated and used as an intraday variation. This experiment was performed for two more days. The average values obtained for the three consecutive days were used as an intraday variation. The relative standard deviation (RSD) values of the corresponding peaks are shown in Table 3. The maximum standard deviation was 2.71%.

**Table 3:** Precision, robustness, and accuracy of TEA, DEIPA, and TIPA tested 6 times per day for three consequent days.

Analytes	Precision (relative standarddeviation, RSD, %)		Robustness(RSD, %)	Accuracy (n = 6)		
	Intraday(n = 6)	Interday(n = 6)		Meanµg/mL	Recovery%	RSD%
TEA	0.94	1.11	1.38	224.63	95.5	1.88
DEIPA	1.33	0.67	1.79	246.34	100.8.	1.43
TIPA	2.71	1.06	0.95	164.89	97.6	0.76

### 3.6. Sample stability during storage

To assess the storage stabilities, the samples were stored prior to the HPLC analysis at room temperature for at 0, 24, and 48 h. The maximum RSD was 1.79%, which indicates the samples can be stored for at least 2 days prior to their analyses.

### 3.7. Accuracy 

To evaluate the accuracy of the developed method, we performed recovery tests. For this purpose, a test sample, as well as a baseline sample, were analysed by HPLC-ELSD. The test sample is prepared by adding a standard solution of the analytes to an aliquot of a cement grinding aids sample solution, and the baseline sample is prepared by adding an equal amount of the mobile phase to the same cement grinding aids sample solution. The alkanolamine amounts in these samples were obtained from the calibration curves.The recovery was calculated using Equation (1).

Recovery (%) = [(M_3_–M_2_) /M_1_] × 100% (1)

Here, M_3_ is the amounts of the test sample determined by HPLC-ELSD method; M_2_ is the amounts of the baseline sample determined by HPLC-ELSD method, and M_1_ is the standard amounts added to the test sample. 

The recoveries for TEA, DEIPA, and TIPA were in the 95.5%–100.8% range, with RSD < 1.88%. Thus, the method developed in this work is accurate and not affected by the matrix. 

## 4. Conclusion

This new and improved method using HPLC-ELSD with a simple gradient elution mode was developed for determining concentration of TEA, TIPA and DEIPA in liquid cement grinding aids, and the selectivity, linearity, accuracy, precision, and stability were assessed. The main advantage of the developed procedure compared to the earlier works is this method requires no complex pretreatment, derivatisation steps or expensive instruments. The method is fast, sensitive and precise, and can be applied for routine quality assurance and product improvements during cement production.
